# Operational and Material Causes of High-Pressure Turbine Disc Damage in the RD-33 Engine

**DOI:** 10.3390/ma16175939

**Published:** 2023-08-30

**Authors:** Stanisław Jóźwiak, Adam Kozakiewicz, Stanisław Kachel, Dariusz Zasada

**Affiliations:** 1 Faculty of Advanced Technology and Chemistry, Institute of Materials Science and Engineering, Military University of Technology, 00-908 Warszawa, Poland; stanislaw.jozwiak@wat.edu.pl (S.J.); dariusz.zasada@wat.edu.pl (D.Z.); 2 Faculty of Mechatronics, Armament and Aerospace, Institute of Aviation Technology, Military University of Technology, 00-908 Warszawa, Poland; adam.kozakiewicz@wat.edu.pl

**Keywords:** turbine jet engine, material tests, ember-resistant alloys

## Abstract

This paper presents an analysis of the causes of damage and fragmentation to the high-pressure turbine (HTP) disc of the RD-33 engine mounted in the MIG-29 aircraft. The authors have carried out an analysis of the changes to the structure of the disc material, both in the areas containing cracks and in the undamaged areas. The impact of structural changes on the alterations in the analysed strength properties along the disc radius was assessed. Material tests were correlated with the analysis of the recorded engine parameters, indicating potential causes of the HPT disc fragmentation.

## 1. Introduction

An analysis of the causes of aviation accidents related to the MiG-29 aircraft operated in Poland has indicated that 9% of these failures resulted from damage to the RD-33 engine [1]. A vast majority of these events were caused by foreign object damage (FOD), leading to the destruction of both the low- and high-pressure compressor blades. Another significant problem, constituting 13% of RD-33 engine failure cases, is damage to the high-pressure turbine (HPT) blades. The existing literature analyses [2,3,4,5,6,7] indicate that the main cause of damage to these engine components are thermomechanical loads facilitated by the erosive and corrosive effect of exhaust gases. In addition, there have been two cases of stage IV fatigue cracks of the fan disc, the indirect cause of which was the extension of the service life of the engines from the original 1200 h to 1600 h of operation, leading to mechanical damage and resulting in the initiation and propagation of fatigue cracks [1].

Extending the service life of engines entails an increase in the number of their start-ups, take-offs, and landings, as well as an extension of their operating time in the ranges characterised by the maximum temperature of exhaust gases, which increases the number of engine work cycles. These factors have a huge impact on the structural changes in the materials of the engine components exposed to high temperatures and may lead to a reduction in the general strength properties, even causing the destruction of individual components. The engine components that are most exposed to thermomechanical loads undoubtedly include the HPT turbine blades and disc. In the case of the RD-33 engine, to a certain, limited extent, diagnostics related to the turbine disc blades are possible in operating conditions, although the analysis of its condition can only occur only after engine disassembly during an overhaul. Therefore, turbine discs must be designed in such a way as to exclude the possibility of their failure, which, in extreme cases, leads to defragmentation due to the possibility of aircraft damage caused by the huge kinetic energy found in the fragments torn from the disc. The modern disc design process is focused on rotating elements exposed to maximum stress, such as the turbine blades root and the turbine disc rim. This means that damage may be expected in these areas. The impact of changes in the microstructure caused by thermal and mechanical loads during engine operation is also significant [8,9]. One study [3] states that after exceeding the limit temperature (even for a short period of time), there is a rapid deterioration of the basic heat resistance of the material, leading to its destruction even after 200 h of operation. However, by increasing the operating temperature by 200 °C above the limit, the material can fail even within 1 h at the same load. The reason for this behaviour of the material is the expansion of grains in the γ’ superstructure, the limit size of which, ensuring the appropriate strength parameters, has been set at 12 µm [10]. In order to prevent the expansion of the γ’ phase grains, the chemical composition of heat-resistant nickel superalloys is modified with additives, which are designed to create carbides within the grain boundaries of the matrix, inhibiting the expansion of the strengthening phase. Nevertheless, the greater chemical activity of these elements can lead to unfavourable phenomena, such as the formation of oxide precipitates within the grain boundaries of the matrix, which is conducive to the initiation and propagation of fatigue cracks [11].

**Figure 1:**
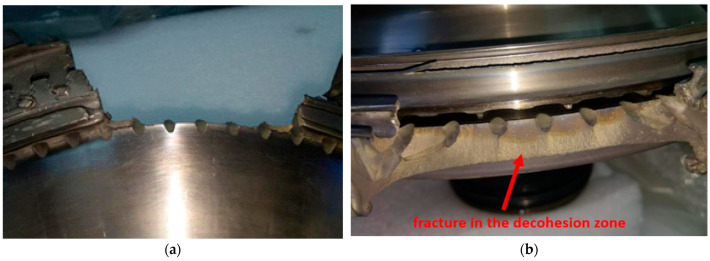
Area where a fragment of the HCP turbine disc of the RD-33 engine has been torn out (**a**), with a view of the resulting fracture in the material decohesion zone (**b**).

Failure to meet the above material and strength conditions may lead to exceeding the permissible level of stress, leading to decohesion of the turbine disc material, resulting in its defragmentation and, therefore, the destruction of the engine (Figure 1).

In this work, changes in the structure of the disc material were analysed, both in the areas of the resulting damage and in zones free of deformations and cracks, while assessing the impact of structural changes on alterations in the strength properties occurring along the disc radius. The material tests were supplemented with an analysis of the course of the changes in the recorded exhaust gas temperature in the tested turbine.

## 2. Test Results and Discussion

### 2.1. Structural and Strength Analysis

**Figure 2:**
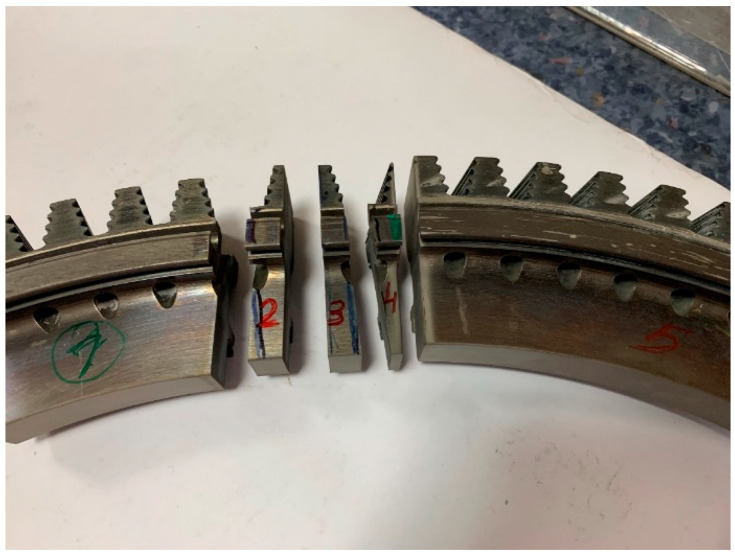
A view of the non-damaged part of the disc cut into pieces with markings of the respective samples.

**Figure 3:**
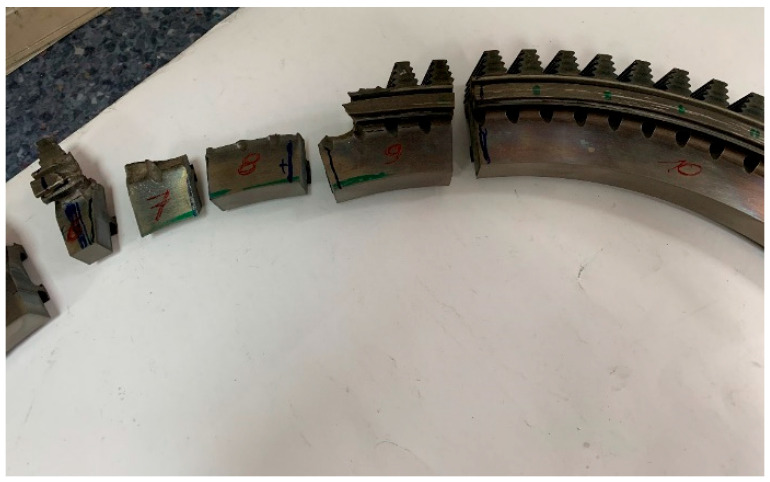
A view of the damaged part of the disc cut into pieces with markings of the respective samples.

Before proceeding with the basic macroscopic examinations, an element of the turbine disc rim cut out from the damaged area was subjected to a preliminary inspection, based on which the areas for further material tests were selected. In order to obtain samples for metallographic tests, the disc was cut into smaller fragments using a plasma cutter. The samples were successively marked with the numbers one to five in the undamaged part of the disc (Figure 2), while in the damaged area, they were marked with the numbers six to nine (Figure 3).

**Figure 4:**
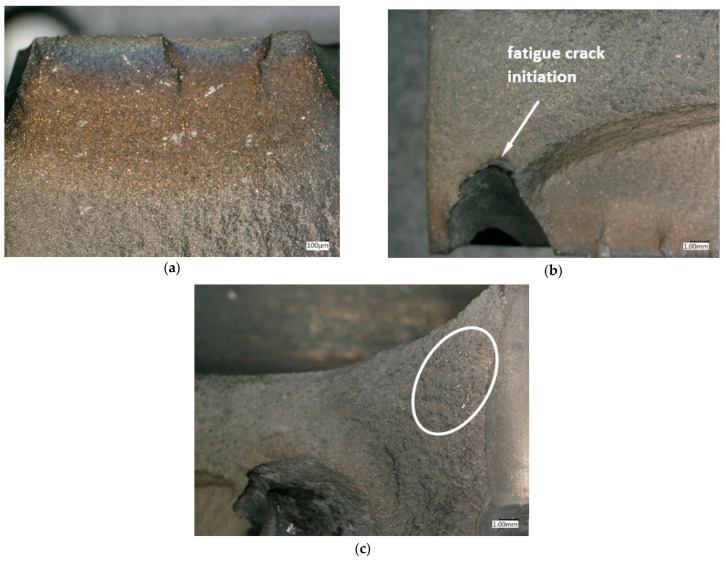
Macroscopic image of the fracture with visible traces of gaseous corrosion (sample 7) (**a**) and the site of fatigue cracking (sample 9) (**b**) in the area of the cooling holes, with a view of bright non-metallic precipitates (sample 9) (**c**).

Macro examinations (Figure 4) carried out with the use of a Keyence VHX-950F digital microscope in the area of disc rim damage allow the conclusion that the damage to this element of the engine was progressing over a long period of time and was not of a temporary nature. This is evidenced by the surface of the analysed fractures without zones of obvious plastic deformation and by the areas which, despite strong oxidation, are characterised by the occurrence of local sites typical of material fatigue damage. Additional evidence confirming this thesis is the observation of discolourations on the surfaces of the analysed fractures, with the darkest shade near the disc surface and decreasing discolouration intensity with increasing distance from the surface. The strongest discolouration starts in the zones where the holes supplying cooling air to the blade interior penetrate the front surface of the turbine disc (Figure 4a). These areas contain morphological changes typical of fatigue sites, where fatigue damage of the material is initiated and crack propagation develops (Figure 4b). A characteristic feature of the analysed fractures is also the occurrence of clusters of bright spots of precipitation, which may constitute non-metallic inclusions in the material structure of the analysed disc (Figure 4c).

**Figure 5:**
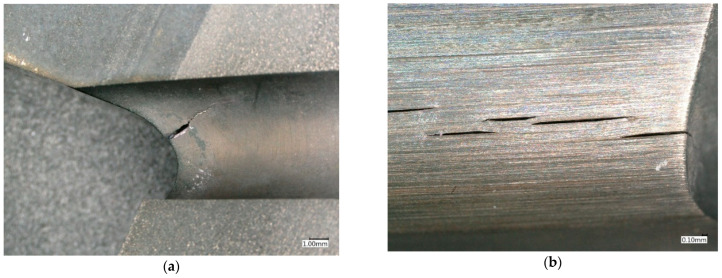

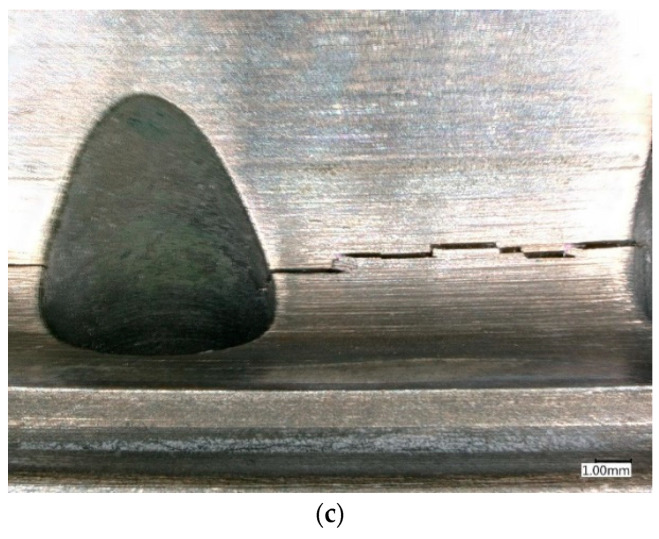
Crack initiated on the surface of the cooling hole in the “undamaged” area of the disc (sample 9) (**a**) with visible propagation in the zone between the holes (**b**), leading to crack joining and material decohesion (**c**).

Macroscopic observation of the disc in the undamaged zone has confirmed the above assumptions. It was found that cracks initiated on the surface of the cooling channels at the front surface of the disc progressed around the periphery in the areas between the holes and then combined in this area, leading to complete decohesion of the disc material (Figure 5).

**Figure 6:**
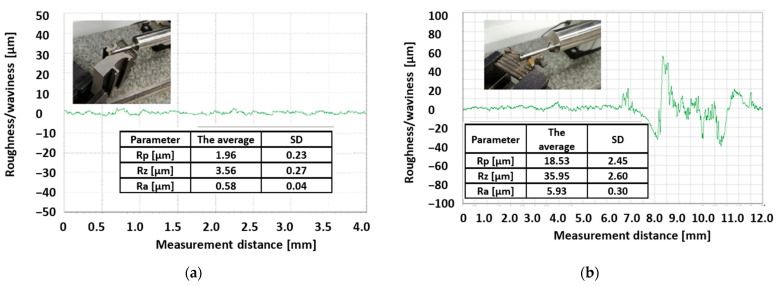
Roughness measurement route in the “undamaged disc” area (**a**) and in the fracture zone formed in the cooling hole area (**b**) with sample results.

The conclusion regarding the long-term fatigue damage of the turbine disc material in the area of the cooling channels in the turbine disc rim area is confirmed by the analysis of the geometric structure of the channels’ surface using the TOPO-01 profilographometer (IOS, Krakow, Poland). The visible effects of the material removal processing on the surface of the channels in the undamaged areas of the disc are characterised by roughness parameters at a level of Rz = 3–7 µm and Ra = 0.3–0.7 µm (Figure 6a). On the other hand, in the fracture developing on the surface of the channel in sample six, these parameters reached the values of Rz = 39.95 µm and Ra = 5.93 µm (Figure 6b).

In order to unambiguously determine the causes and nature of damage to the material of the tested turbine disc, the following examinations were performed: structural and fractographic tests, assessment of wear traces and chemical composition of this structural element, and assessment of other selected elements of the analysed turbine assembly, such as blades, dampers, and the retaining ring. These analyses were carried out using a Quanta 3D FEG (SEM/FIB) (FEI Company, Hillsboro, OR, USA) high-resolution scanning electron microscope equipped with an integrated EDS/WDS/EBSD system (EDAX, Inc. Mahwah, NJ, USA) (EDS—energy dispersive X-ray spectroscopy, WDS—wavelength dispersive X-ray spectroscopy, EBSD—electron backscatter diffraction).

The structure of the appropriately prepared metallographic micro-sections collected from the areas of observed cracks and undamaged zones has been comprehensively examined. The fractures have also been assessed using samples collected from selected areas of the damaged turbine.

**Figure 7:**
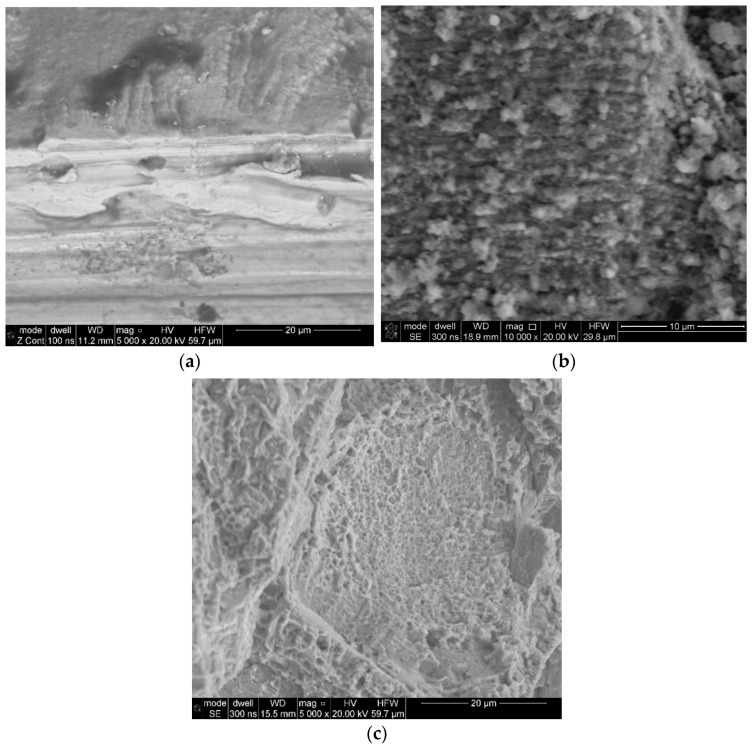
Localised fatigue site in the cooling channel area (**a**), fatigue crack propagation zone with characteristic fatigue bands (**b**), and fracture area with visible traces of a plastic fracture (**c**).

Fractographic studies of the surfaces were carried out using samples collected from the “damaged” area of the turbine before and after cleaning the analysed surface in an ultrasonic cleaner with the use of acetone. Based on these observations, it was found that the analysed fragments demonstrated characteristic features of a fatigue fracture. Numerous fatigue crack origin sites were observed by the fracture surface (Figure 7a). Each time, fatigue bands (Figure 7b) and a residual fracture zone (Figure 7c) were observed in the fatigue area above the fatigue sites.

**Figure 8:**
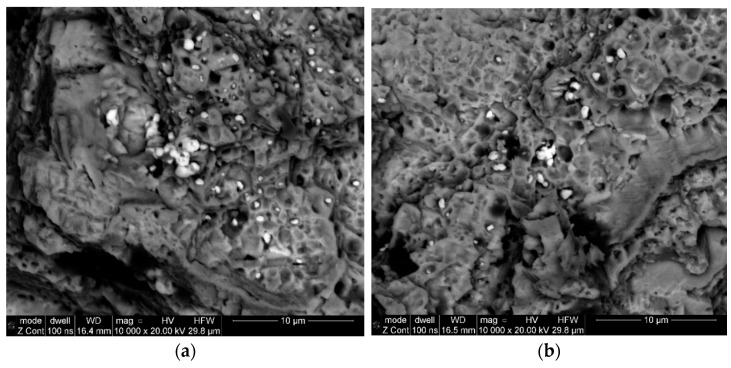
Concentrations of non-metallic precipitates in the area of residual fracture, sample 6 (**a**) and sample 7 (**b**).

**Figure 9:**
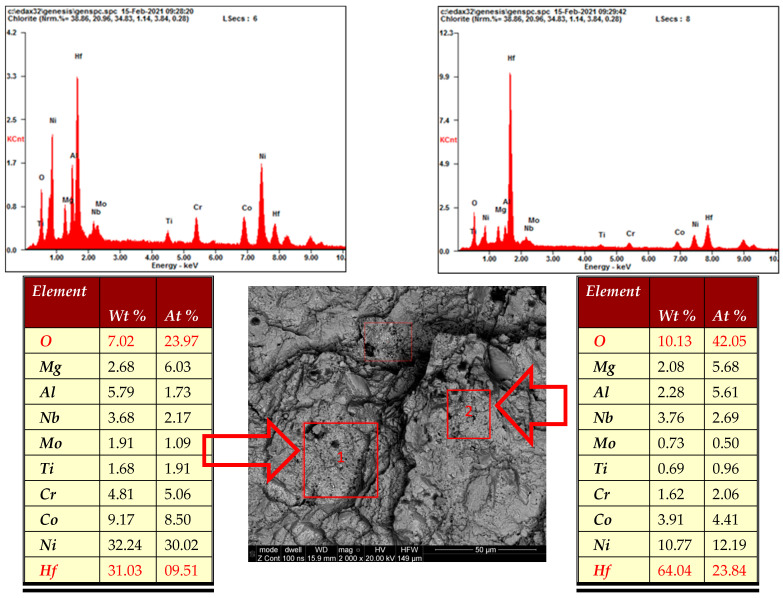
Spectrum EDS and qualitative analysis of non-metallic precipitates identified in the fracture zone. “1” and “2”—areas subjected to analysis of chemical composition using the WDS method.

**Figure 10:**
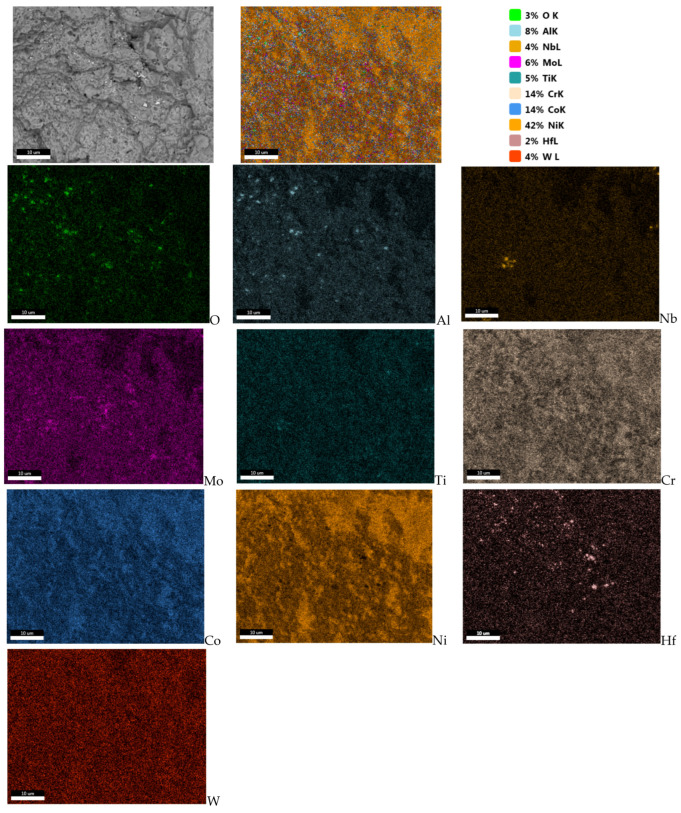
Surface distribution of elements in the residual fracture zone, showing clusters of hafnium and aluminium oxides.

In addition, observations of fractures in the crack fracture zone revealed the presence of numerous clusters of fine non-metallic precipitates (Figure 8a). The point analyses of the chemical composition of samples collected from specific areas demonstrated that they were hafnium oxides (Figure 9). The confirmation of this observation is the surface distribution of elements shown in Figure 10, which, apart from identifying hafnium oxides, also allows for observation of the occurrence of aluminium oxide spots.

**Figure 11:**
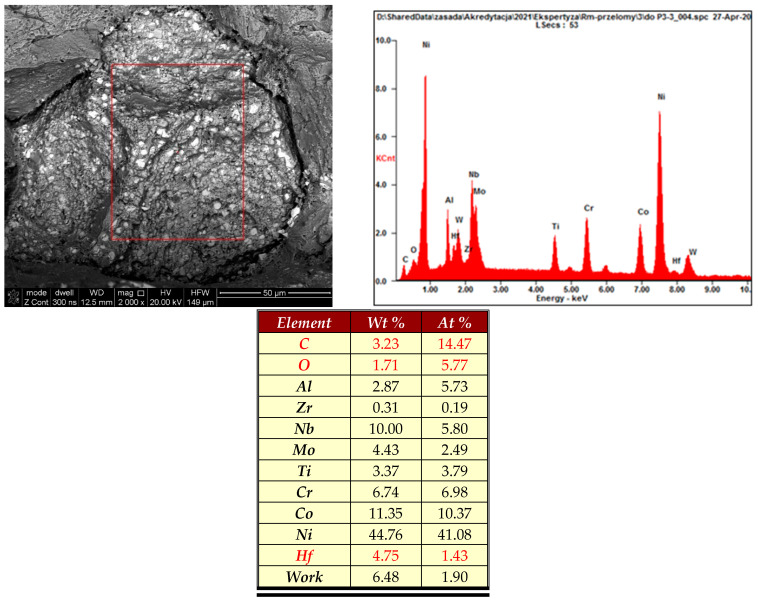
Spectrum and results of the qualitative analysis of the chemical composition in the area marked with a red square, containing complex carbide-oxide inclusions identified in the fatigue cracking zone.

In addition, in the fatigue fracture zones, we identified areas of about 50 µm made of complex oxide carbide structures (Figure 11), which were undoubtedly structural notches in the analysed alloy volume. These undesirable precipitates could not have formed during thermal processing or exploitation, which suggests that they were formed in the structure of the material during the metallurgical process of alloy production.

**Figure 12:**
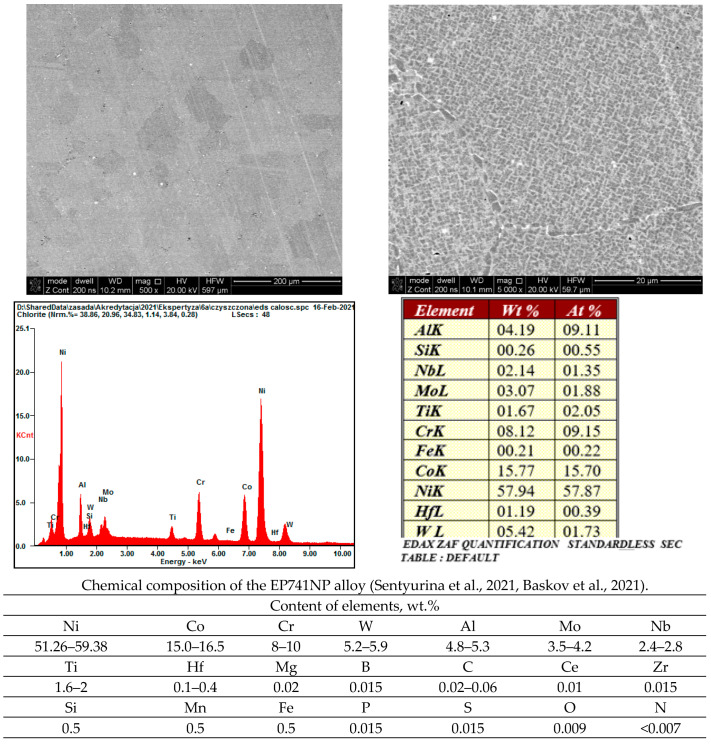
Two-phase γ + γ’ structure of the tested alloy with carbide precipitations within grain boundaries, with an analysis of the chemical composition and literature data [13,14] regarding the composition of the EP741NP alloy.

In order to unambiguously link the observed fatigue damage of the turbine disc material to the grain structure of the alloy used, microscopic observations were carried out, which clearly demonstrated that the material from which the turbine disc was made had a grain structure typical of the heat-resistant nickel superalloy produced using the powder metallurgy process. The observation of metallographic micro-sections revealed that the tested alloy was composed of primary γ grains containing cubic γ’ phase precipitations (Figure 12). It has been estimated that the size of the primary γ areas is in the range of 50–150 µm. Along the boundaries of the observed primary grains, there are overgrown precipitates of the γ’ phase and carbide precipitates preventing grain growth and high-temperature creep. The microanalysis of the chemical composition of the tested material corresponds to the Russian EP741NP superalloy used for the construction of turbine discs in high-load turbine jet engines [12].

**Figure 13:**
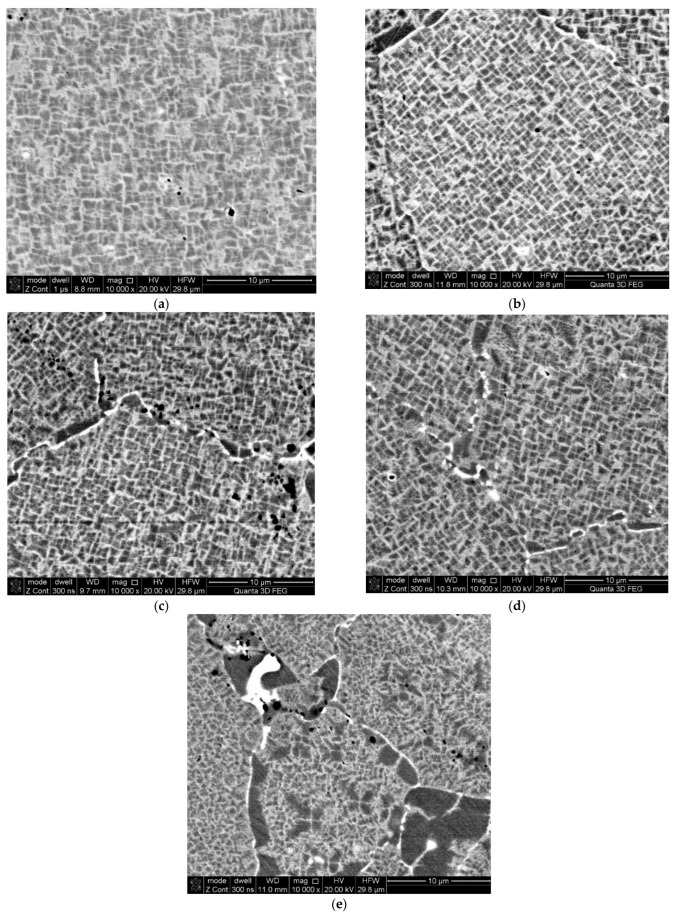
Changes in the cubic grains of the γ’ phase in the alloy matrix and the growth of γ’ and carbide precipitates within the boundaries of the original γ grains of the examined EP741NP alloy in the areas of samples collected for strength tests along the disc radius; distance from the foot plate of the disc: 23 mm (**a**), 63 mm (**b**), 85 mm (**c**), 128 mm (**d**), and 175 mm (**e**).

Nevertheless, due to the observed changes in strength properties along the disc radius and due to the identified fatigue cracks, it was necessary to carry out observations in these areas. Changes in the grain structure along the disc radius were analysed first (Figure 13).

Based on the microscopic observations, it was found that the grain structure of the alloy in the area of the turbine disc rim (Figure 13a), i.e., in the zone of maximum thermal impact of hot exhaust gases, revealed clear differences in the morphology of the grain structure compared to the areas located below the rim (Figure 13b–e). A clear, selective growth of cuboidal grains of the γ’ phase can be observed, leading to strong disorientation within the primary grains of the solid γ solution. In addition, within the original boundaries, the effects of anomalous growth of the γ’ superstructure and carbide precipitates were observed. Such a morphological alteration is conducive to the development of cracks in the material between disoriented, expanded precipitates of the γ’ phase, both along the primary grain boundaries and transcrystalline [15,16].

**Figure 14:**
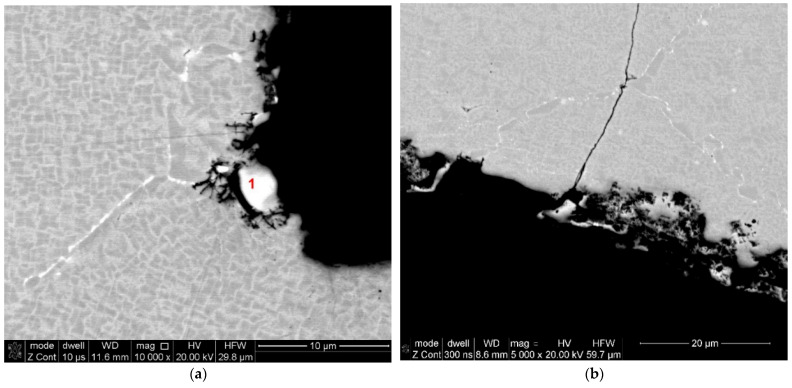
Crack origin in the primary grain boundaries of γ solid solution in the area of γ’ phase grains and carbide precipitates marked with the number 1 (**a**) and crack propagation between γ’ phase grains (**b**).

The cause of these structural changes is the long-term impact of high temperatures on the alloy structure. Literature reports on temperature changes in the structure of the EP741NP alloy and their impact on changes in mechanical properties, including mainly high-temperature creep resistance, clearly indicate that exceeding the total operating time of more than 200 h at a temperature of 750 °C causes structural changes, which were also observed in our study, leading to a decrease in strength properties [16,17,18,19]. Due to the observed structural component alterations, the structure in the crack areas was assessed. Sample six, from the damaged area of the disc, confirmed the literature reports that the fatigue cracks originated in overheated areas of primary grain boundaries of γ solid solution characterised by overgrown grains of the γ’ phase and carbide precipitates (Figure 14a) and propagating between the grains of the γ’ phase (Figure 14b), which confirms the cracking mechanism described in the works [15,16].

**Figure 15:**
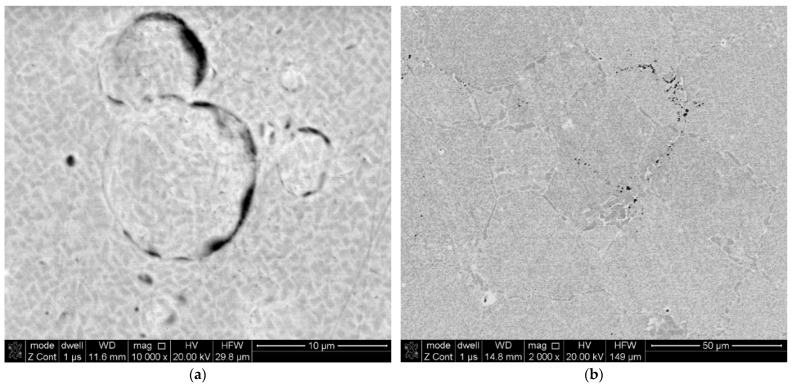
Oxidised, primary powder particles unreacted during the sintering process (**a**) and numerous ceramic precipitates within the primary grains of the matrix (**b**).

When analysing the observations of the tested alloy microstructure, it is impossible to ignore the identified hafnium oxide precipitates (Figure 9 and Figure 10). According to literature reports, this element was added to the EP741NP alloy to form hafnium carbides, limiting the growth of other M_23_C_6_ and MC carbides within the grain boundaries and thus improving the resistance to high-temperature creep [20,21]. However, according to the results presented in [22], failure to maintain the technological process regime at the alloy production stage may lead to the formation of hafnium oxide (HfO_2_) precipitates with a monoclinic structure, significantly contributing to the development of fatigue cracks, which was also confirmed in the case of the examined disc material. It should also be noted that, in addition to the previously found and described material defects in the structure of the analysed alloy, in the area of fatigue crack origin, primary particles of the powder used in the disc material sintering process were observed, which were covered with oxide impurities, which prevented the proper occurrence of the diffusion processes and obtention of a homogeneous sinter (Figure 15a). In addition, numerous oxides and nitrides were identified in the areas of the primary grain boundaries of the solid γ solution, weakening the cohesion and strength of the boundaries (Figure 15b).

**Figure 16:**
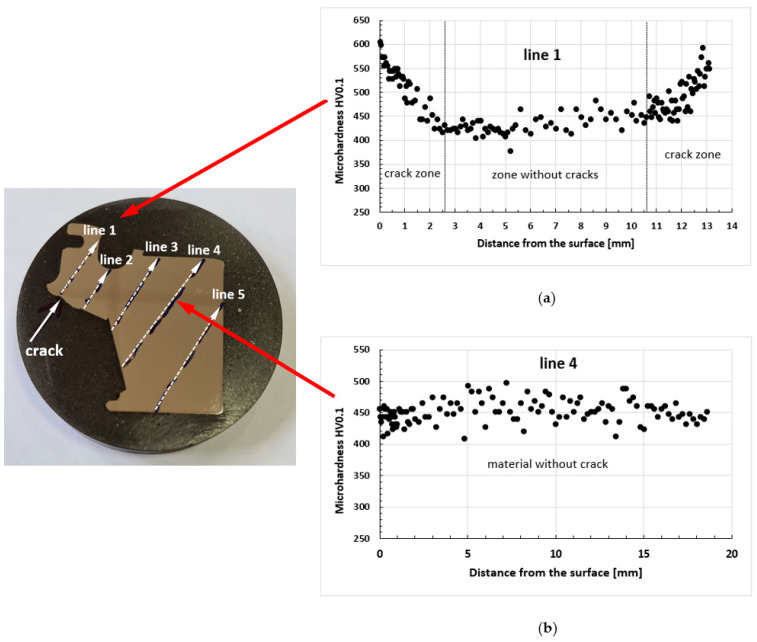
Microhardness distribution along line 1 in the area of crack propagation (**a**) and along line 4 in the zone of homogeneous material (**b**).

The growth of carbide phases, which are the direct cause of the initiation and propagation of fatigue cracks (see Figure 13e and Figure 14a), was also confirmed by the microhardness measurements (Figure 16). They demonstrated homogeneous distribution of this parameter value at the level of 450HV0.1 in all undamaged areas (Figure 16b), regardless of their location as a function of the disc radius, indicating the macroscopic homogeneity of the material. On the other hand, a noticeable increase in microhardness, to the level of 600HV0.1, was observed in the area of fatigue crack propagation (Figure 16a).

**Figure 17:**
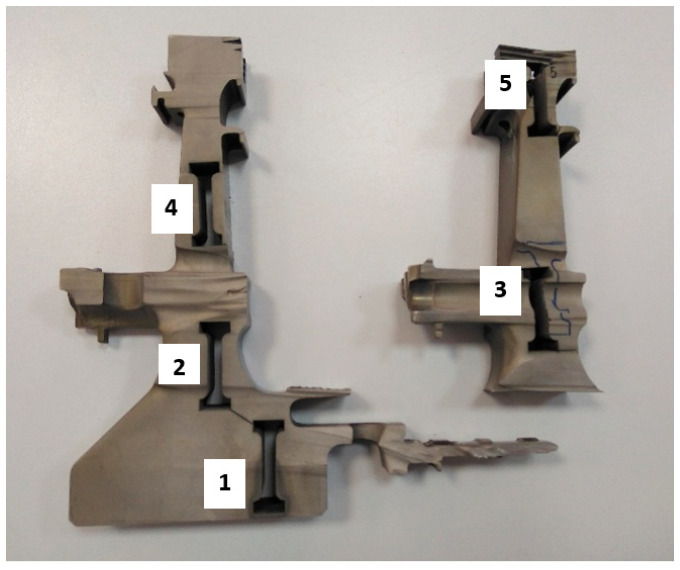
The location of sample collection from sections of the turbine disc for the static tensile test. “1–5”—areas of cutting strength samples described in the text.

The structural changes of the material observed along the disc radius (Figure 13) should also be reflected in the mechanical properties. In order to determine the basic, static strength parameters in the disc sections (Figure 17), tensile test samples were collected from five areas: one—disc foot plate, two—disc zone between the labyrinth seal and the connecting drum, three—drum zone, four—zone between the blade rim and drum part, and five—retaining ring area. The prepared samples were subjected to tension on an Instron 8501 testing machine using an extensometer with a measuring length of 12.5 mm and TestXpert III v.1.5 software. The tests were carried out in accordance with the requirements of the PN-EN ISO 6892-1:2020-05 standard [23].

**Figure 18:**
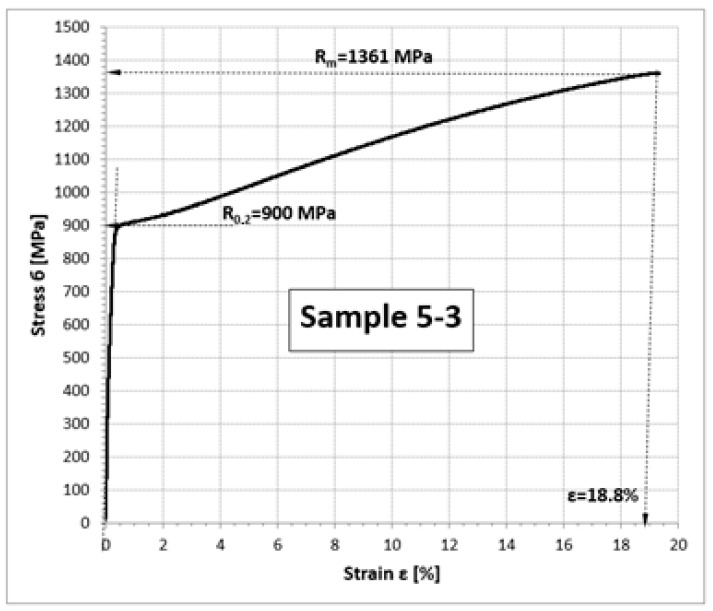
Sample stress–strain curve obtained for a sample collected from the retaining ring zone.

As a result of the test, stress–strain curves (б-ε) (Figure 18) were obtained based on which the basic strength properties of the tested samples were determined, including: yield strength R_p0,2_, tensile strength R_m_, and relative elongation A (Table 1).

**Figure 19:**
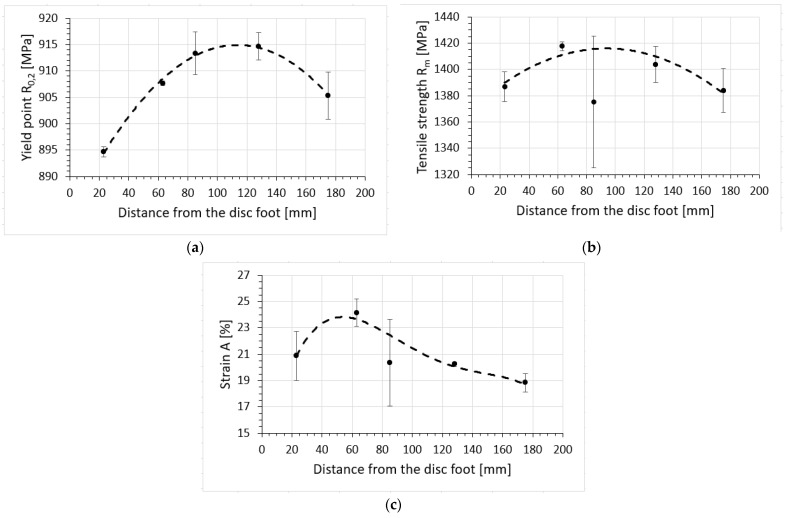
Changing the yield point R_0.2_ (**a**), tensile strength R_m_ (**b**) and strain A (**c**) of the turbine material as a function of the distance from the disc base.

The changes in the determined average strength parameter values along the line running from the turbine foot plate are shown in Table 2 and Figure 19.

The strength properties of the turbine disc material, determined by means of a static tensile test, show a non-monotonic change in the strength properties along the disc radius, with the maximum value of R_0.2_ and R_m_ for the distance from the disc foot plate in the range of 60–130 mm, i.e., in the zone of maximum cooling, with a noticeable decrease in plasticity in the rim area, i.e., the maximum temperature impact area. In addition, it should be noted that the values of strength and plastic properties determined at ambient temperature are lower than those reported in the literature [16,24] for the EP741NP alloy of the 1995 generation, while meeting the requirements for the EP741NP alloy of the 1981 generation (Table 3).

### 2.2. Analysis of the Engine Operating Parameters

Material tests were correlated with the analysis of the recorded engine parameters, such as exhaust gas temperature behind the turbine. They indicated the potential causes of HTP disc fragmentation.

**Figure 20:**
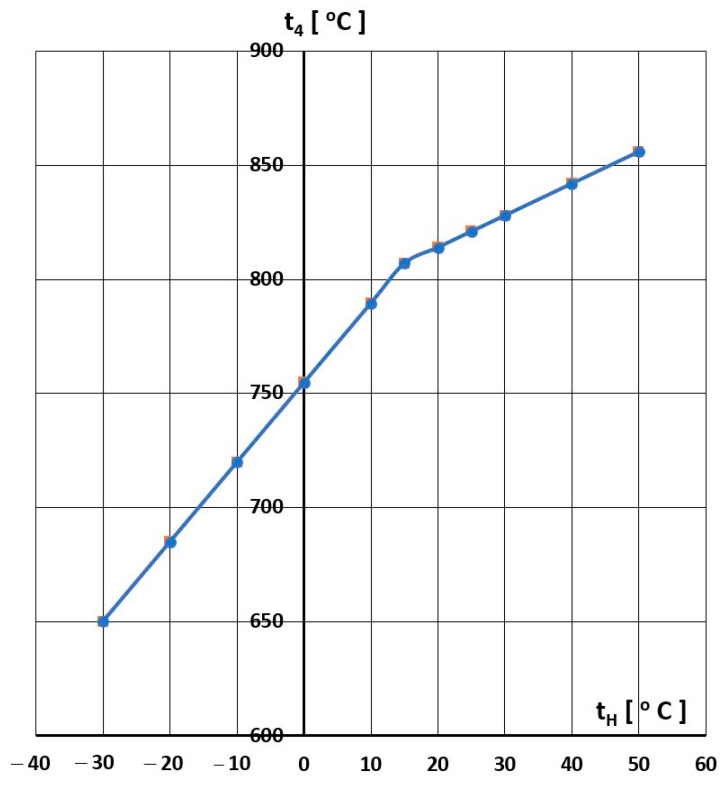
Temperature limit curve t4 for the tested engine.

In the case of the RD-33 engine, its operating manual defines the permissible operating temperatures of the engine in the turbine area by means of the temperature behind the turbine. Based on the manual, the temperature change behind the turbine $t_{4}$ was characterised as a function of the temperature at the engine inlet${\;t}_{H}$, which is shown in Figure 20. This temperature characteristic results from the engine control program based on the temperature behind the turbine in relation $t_{4}$ to the temperature before the turbine combined with compressor maps, which depends on the temperature of the air entering $t_{H}$ the engine and the rotational speed. The characteristic $t_{4}=f\left(t_{H}\right)$ involves two ranges of engine operation (Figure 20) as a function of ambient temperature, the first from −30 °C to +15 °C and the second from +15 °C to +50 °C.

**Figure 21:**
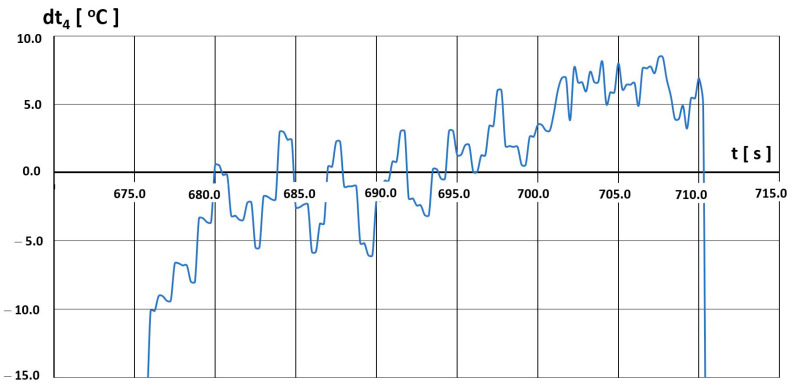
Temperature t_4_ deviations from engine characteristics in the time interval (680–710) s.

**Figure 22:**
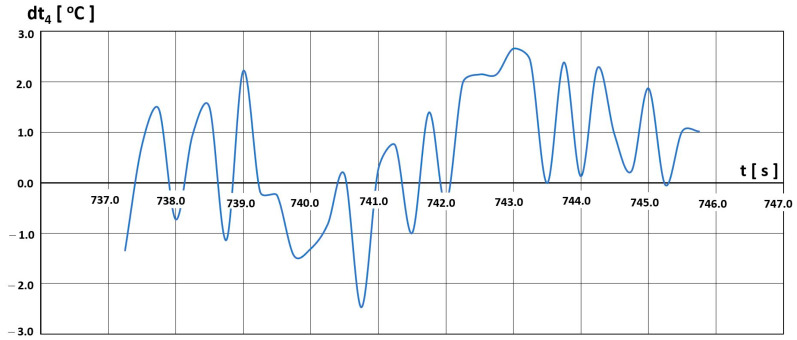
Temperature t_4_ deviations from engine characteristics in the time interval (737–746) s.

**Figure 23:**
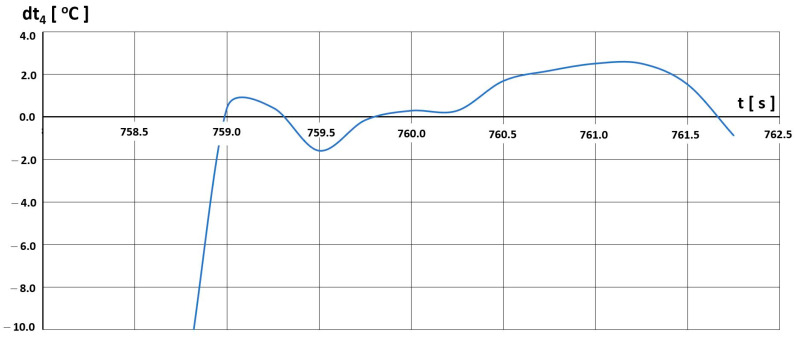
Temperature t_4_ deviations from engine characteristics in the time interval (758–762) s.

After determining the characteristics$\;t_{4}=f(t_{H})$, we analysed the selected flight parameter changes. The temperature $t_{4}$ was assessed in terms of the possibility of exceeding the limit values. Sample temperature value differences $dt_{4}$ between the permissible value ${\;t}_{4,D}$ resulting from the limitations imposed by the manufacturer (Figure 20) and the value recorded in the engine ${\;t}_{4,L}$ are shown in Figure 21, Figure 22 and Figure 23.

Three intervals of excessive temperature$\;dt_{4}$, i.e., temperature exceeding the permissible limit for this engine, have been established:

- First—(680–710) s—Figure 21;
- Second—(737–746) s—Figure 22;
- Third—(758–762) s—Figure 23.

In the first interval (Figure 21), we observed excess temperature values of even up to $dt_{4}=8\;{}^{\circ}C$. In the initial period, from 680 s, the exceedance was of a temporary nature, maintaining more or less the same value in the order of 3 °C. After 15 s, they assumed the character of a continuous exceedance, with the value increasing for 16 s. Temperature exceedance subsided at 711 s.

The second interval (Figure 22) is characterised by a different course of the temperature exceedance $dt_{4}$ compared to the first one. The event was not continuous and had an oscillatory character. Exceedances of 2.5 °C are achieved, and the time of the entire interval lasted about 8 s.

The third interval (Figure 23) had a completely different course of temperature exceedance. At 759 s, there was a slight exceeding of the permitted limit temperature, which then became continuous in nature, lasting about 12 s, and then reaching a value of up to 3 °C.

This type of exceeding the permitted limit temperature, due to its cyclical nature, may cause turbine strength reduction by increasing the load of this assembly in terms of the thermal stress and low-cycle fatigue. These types of loads are important in the long term because of their cumulative effect, which, in the analysed case, led to the destruction of the tested engine subassembly, the high-pressure turbine disc.

## 3. Summary and Conclusions

As a result of the structural and strength tests of the material of the damaged disc of the RD-33 engine, it can be concluded that:

The chemical composition of the examined disc lets us unequivocally state that it is type EP741NP Russian-made heat-resistant nickel-based alloy.

The phase structure of the alloy used in the examined disc production corresponds to the typical structure of heat-resistant nickel-based superalloys and consists of a two-phase γ + γ’ matrix with carbide precipitations.

The morphology of the grain structure allows us to clearly state that the examined turbine disc was made using the powder metallurgy process.

In the entire volume of the material structure of the examined disc, local areas with the occurrence of clusters of hafnium oxide and titanium nitride spots were observed, which were classified as a 0.5 purity class according to the Polish Standard PN-64/H-04510 [25].

In the area of the blade rim, the effects of the strong, selective growth of the γ’ phase were observed, both in the area of grain boundaries and in the matrix, as well as intensive growth of the carbide phase in the areas of grain boundaries, caused, according to the literature data, by the effect of long-term, over 200 h long periods of continuous exposure to temperatures above 750 °C, causing overheating of the turbine disc material in this area.

The strength properties of the turbine disc material, determined by means of a static tensile test, show a non-monotonic change in the strength properties along the disc radius, with the maximum value of R_0.2_ and R_m_ for the distance from the disc foot plate in the range of 60–130 mm, i.e., in the zone of maximum cooling, with a noticeable decrease in plasticity in the rim area, i.e., the maximum temperature impact area.

The values of the strength and plastic properties determined at ambient temperature are lower than those reported in the literature [14,15] for the EP741NP alloy of the 1995 generation while meeting the requirements for the EP741NP alloy of the 1981 generation.

The Vickers microhardness measurements carried out with a load of 100 G demonstrated a uniform distribution of the parameter value of 450HV0.1 over the entire surface of all the tested areas, indicating the macroscopic homogeneity of the material of the tested samples. A noticeable increase in microhardness up to the level of 550-600HV01 was observed in the area of fatigue crack propagation.

Numerous fatigue cracks, characterised by changes in the morphology of the grain structure, were observed in the rim area. The analysis of fracture morphology, both in the uncleaned state and after cleaning, reveals numerous fatigue bands propagating into the disc material from the foci located on the surface in the cooling hole area. The crack development follows a typical mechanism characteristic for heat-resistant nickel alloys produced using the powder metallurgy process along the expanded carbide precipitates within the grain boundaries and between the expanded crystallites of the γ’ phase.

In numerous cases, hafnium oxide clusters were found on the revealed fractures, whose precipitates, according to literature reports, are areas conducive to the origination and propagation of fatigue cracks [22].

The undamaged surfaces of the cooling channels in the area of the disc rim have visible effects of machining, characterised by the roughness parameters Rz = 3–7 µm and Ra = 0.3–0.7 µm.

No effects of high-temperature oxidation were observed on the surfaces of the cooling channels, which proves the correct flow of cooling air during the disc operation.

In the case of the RD-33 engine, its operating manual defines the permissible operating temperatures of the engine in the turbine area. Based on the manual, a graph of the temperature change behind the turbine (t_4_) was made as a function of the temperature at the entrance to the engine (t_H_) and the behaviour of the exhaust gas temperature (t_4_) during the flight of the examined engine to check for possible exceedances. As a result of the analysis, three types of the permitted limit temperature excess were obtained. In the first type, there were excess temperature values of even up to 8 °C. In the initial period, the exceedances were of a temporary nature, maintaining more or less the same value. They were of a continuous nature with an increasing value. The second interval is characterised by a different course of the temperature exceedance compared to the first one. The event was not continuous and had an oscillatory character. The temperature exceedance reached a value of 2.5 °C, and the entire event lasted for about 8 s. The third interval has a completely different course of temperature exceedance. There was a slight exceeding of the permitted limit temperature, which then became continuous in nature, lasting about 12 s and then reaching a value of up to 3 °C. This type of exceeding the permitted limit temperature, due to its cyclical nature, may cause turbine strength reduction by increasing the load of this assembly in terms of the thermal stress and low-cycle fatigue.

## Figures and Tables

**Table 1:** Dimensions and determined strength properties of the tested samples.

Sample Specification	Thickness [c] [mm]	Width [mm]	Yield Strength R_p0.2_ [MPa]	Tensile Strength R_m_ [MPa]	Elongation A [%]
1-1	2.43	4.14	896	1391	19.5
1-2	2.47	4.14	894	1371	19.6
1-3	2.44	4.13	894	1398	23.5
2-1	2.43	4.12	907	1421	23.4
2-2	2.45	4.12	908	1419	25.6
2-3	2.43	4.12	908	1413	23.4
3-1	2.45	4.12	911	1423	23.8
3-2	2.46	4.12	910	1306	15.9
3-3	2.45	4.12	919	1397	21.3
4-1	2.41	4.12	911	1423	20.4
4-2	2.40	4.13	917	1392	20.0
4-3	2.43	4.12	916	1396	20.3
5-1	1.76	4.12	905	1391	19.7
5-2	1.75	4.12	911	1400	18.0
5-3	1.76	4.12	900	1361	18.8

**Table 2:** The changes in the average strength parameter values as a function of the distance from the turbine foot plate.

Distance from the Disc Foot Plate [mm]	R_p0.2_ [MPa]	R_m_ [MPa]	A [%]
23 (area 1)	894.7 ± 0.9	1386.7 ± 11.4	20.9 ± 1.9
63 (area 2)	907.7 ± 0.5	1417.7 ± 3.4	24.1 ± 1.0
85 (area 3)	913.3 ± 4.0	1375.3 ± 50.2	20.3 ± 3.3
128 (area 4)	914.7 ± 2.6	1403.7 ± 13.8	20.2 ± 0.2
175 (area 5)	905.3 ± 4.5	1384.0 ± 16.7	18.8 ± 0.7

**Table 3:** Comparison of strength properties obtained during a static tensile test with literature data.

Parameter	Tested Disc Material		EP741NP Generation 1981 [16]	EP741NP Generation 1995 [16]	EP741NP [24]
Rm [MPa]	r = 23	1387	1355	1420	1560
	r = 63	1418			
	r = 85	1375			
	r = 128	1403			
	r = 175	1384			
R0.2 [MPa]	r = 23	895	885	1025	1020
	r = 63	908			
	r = 85	913			
	r = 128	915			
	r = 175	905			
A[%]	r = 23	21	17	20	19
	r = 63	24			
	r = 85	20			
	r = 128	20			
	r = 175	19			

## Data Availability

Not applicable.
